# Retinal pigment epithelium pathology in age-related macular degeneration: mitigation with melatonin

**DOI:** 10.3389/fmed.2026.1813015

**Published:** 2026-04-13

**Authors:** Russel J. Reiter, Ramaswamy Sharma, Janusz Blasiak, Sergio Rosales-Corral, Doris Loh

**Affiliations:** 1 Department of Cell Systems and Anatomy, Long School of Medicine, UT Health, San Antonio, TX, United States; 2 Department of Biomedical Sciences, Baptist University College of Osteopathic Medicine, Memphis, TN, United States; 3 Faculty of Medicine, Collegium Medicum, The Mazovian University in Plock, Plock, Poland; 4 Centro de Investigacion Biomedica de Occidente, Instituto Mexicano del Seguro Social, Guadalajara, Mexico; 5 Independent Researcher, Marble Falls, TX, United States

**Keywords:** AMD, drusen, inflammation, melatonin, mitochondrial dysfunction, oxidative stress, photoreceptors

## Abstract

Approximately 1.5 million Americans over the age of 40 suffer from vision-threatening age-related macular degeneration (AMD), a number expected to rise with aging demographics. AMD exists in two defined forms: dry (non-exudative) which accounts for up to 90% of cases, and wet (exudative). Dry AMD is characterized by the slow buildup of drusen under the retina, eventually leading to geographical atrophy. Wet AMD involves vascular endothelial growth factor (VEGF)-induced blood vessel formation from the choriocapillaris into the subretinal space, a process referred to as neovascularization. These newly formed blood vessels leak fluid into the subretinal space leading to atrophy of the retinal pigment epithelium (RPE) and associated photoreceptors. Despite clinical distinctions, dry and wet AMD share overlapping pathophysiological features, marked by degeneration of the RPE and the overlying photoreceptors. A major feature of the RPE and photoreceptors are their high metabolically activity and their large numbers of mitochondria, which generate reactive oxygen species (ROS) during ATP production. ROS-induced oxidative stress damages lipids, proteins and DNA, resulting in cellular degradation which contributes to AMD. Because of the elevated oxidative stress levels, antioxidants which neutralize ROS are often recommended as a treatment for AMD. A major objective of this review is to examine the role of melatonin, a powerful and multifunctional antioxidant, in altering the trajectory of AMD progression. Melatonin is synthesized in the RPE and photoreceptors of young individuals but its expression declines with age. As shown in an epidemiological report, its loss contributes to age-related degeneration of the RPE and photoreceptors. Moreover, melatonin inhibits VEGF, suggesting that it would be useful as a treatment for wet AMD. This review explores melatonin-mediated protective mechanisms in the retina, a likely mechanistic basis for the already published findings showing that melatonin use by humans is associated with delayed AMD, and potential clinical applications.

## 1 Introduction

The number of patients with vision impairment due to age-related macular degeneration (AMD) increased from 3.64 million in 1990 to 8.06 million in 2021 (1). This is consistent across two independent Global Burden of Diseases (GBD)-linked analyses (2). However, despite increasing absolute case numbers, age-standardized prevalence rates (ASPR) have declined globally, from about 100 per 100,000 in 1990 to 94 per 100,000 in 2021 (1). Global AMD prevalence is projected to increase from 196 million in 2020 to 288 million by 2040, driven largely by population aging (3).

AMD develops through the interaction of genetic predispositions and environmental/lifestyle factors. Variations in complement pathway genes, especially *CFH, C3, C2*, and *ARMS2/HTRA1*, increase susceptibility to chronic inflammation and drusen formation (4, 5). Aging is the primary AMD risk factor and is associated with cumulative oxidative stress-induced injury to the RPE, Bruch’s membrane, and photoreceptors (5). Tobacco smoking promotes oxidative stress and complement dysregulation and is considered the strongest modifiable AMD risk factor (6). Low intake of antioxidants and omega-3 fatty acids increases AMD risk and the Mediterranean-style diets are protective (7, 8). Cardiovascular/metabolic factors, such as hypertension, dyslipidemia, and obesity, are also risk factors for AMD (8, 9).

Dry (atrophic) AMD is responsible for 85–90% of cases (9). The core mechanisms of this form of AMD include chronic RPE dysfunction, drusen accumulation, and geographic atrophy (10). Drusen accumulation between RPE and Bruch’s membrane leads to RPE degeneration, photoreceptor death, and atrophy expansion (geographic atrophy in late stages). Chronic low-grade complement activation and oxidative stress drive progressive RPE cell loss (11). Wet (neovascular/exudative) AMD accounts for 10–15% of cases but causes the most severe vision loss (12). The core mechanism underlying wet AMD is pathological neovascularization due to VEGF-driven angiogenesis. Stress imposed on RPE may cause hypoxia, VEGF overexpression, and choroidal neovascular membranes (CNV) growing through Bruch’s membrane. These vessels are fragile, causing leakage and hemorrhage, leading to rapid photoreceptor damage. This may result in scarring if left untreated. In contrast to dry AMD, which is untreatable, wet AMD is treated with anti-VEGF injections.

This literature survey was undertaken because of the prominent role of oxidative stress in the initiation and progression of both forms of AMD as well as the discovery of melatonin in mitochondria of somatic cells (13–15), including those of the RPE. Melatonin and its metabolites are potent and multifunctional antioxidants which unequivocally reduce oxidative stress in many different *in vitro* and *in vivo* models (16–18). Its antioxidative actions stem from its ability to stimulate antioxidant enzymes, directly scavenge free radicals (reactive oxygen and reactive nitrogen species), bind transition metals that are involved in free radical generation and other actions (19–21). Studies to date showing that melatonin slows the progression or reduces the severity of this debilitating condition in established animal models of this disease and possibly in humans (22, 23).

## 2 Pathology of retinal pigment epithelium during age-related macular degeneration

**Figure 1:**
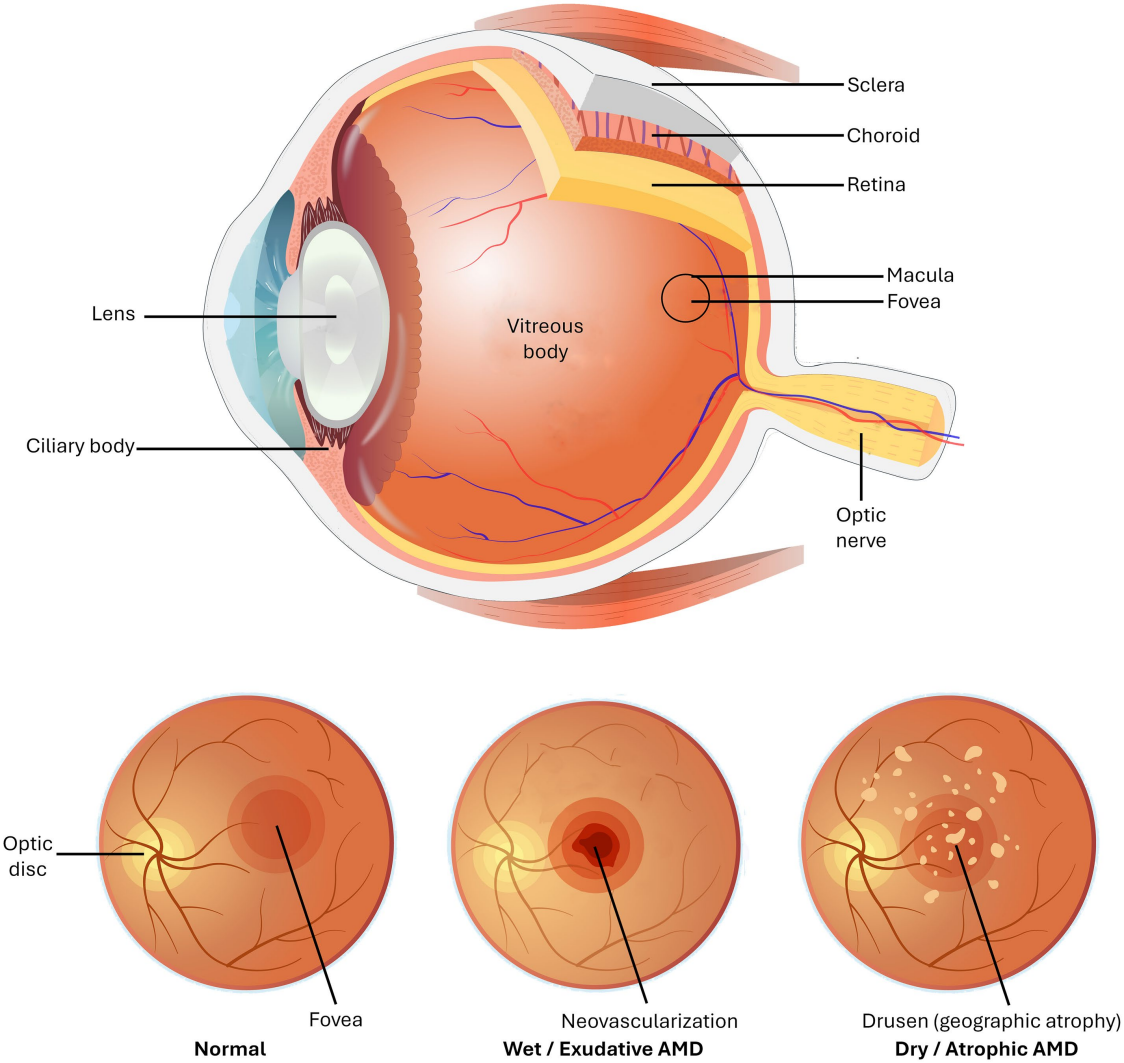
Structure of the ocular globe with the identification of the tissue layers involved in retinal pigment epithelium dysfunction and age-related macular degeneration (AMD) (Top). The three images at the bottom illustrate the appearance of the normal retina, the retina exhibiting features of neovascularization typical of wet AMD, and the retinal appearance common to dry AMD. While multiple drusen develop in the subretinal region and/or in the sub-RPE location and are usually present in the retina of dry AMD, they can also occur in the retina of wet AMD. There are other pathological features of these diseases that overlap the two subtypes. Specifically, how drusen relate to AMD is not established, i.e., whether they are a cause or an effect of the disease is a point of debate.

The RPE, a single layer of polarized cells that is situated between the rods and cones and the choriocapillaris in the outermost portion of the retina, undergoes major pathological changes during AMD progression (24, 25) (Figure 1). RPE cells are typically classified as post-mitotic *in vivo* although a human derived RPE cell (ARPE-19) does proliferate *in vitro*. The RPE consists of specialized cells that are functionally diverse in the maintenance of optimal visual health (26). The numerous essential functions of the RPE encompass the movement of circulating nutrients into and removal of unusable metabolites out of the photoreceptors, maintenance of the visual cycle by reprocessing vitamin A, synthesis of elements that make up the extracellular matrix, secretion of essential growth factors, absorption of stray light for improving image clarity, and phagocytosis of shed outer photoreceptor segments (27). Also, because the RPE cells are connected by tight junctions, this layer forms an important outer blood-retina barrier (oBRB) is primarily an epithelial barrier which includes Bruch’s membrane. It limits potentially harmful agents from entering the retina (28). Bruch’s membrane consists of the basal lamina of the RPE and specialized acellular extracellular matrix that separates the RPE from the choriocapillaris (29). Additionally, the endothelial cells of the choriocapillaris are connected by tight junctions and, along with astroglial end-feet, pericytes, and Müller glial cells, form the inner blood-retinal barrier (iBRB). The iBRB is a vascular barrier the capillaries of which possess non-fenestrated endothelial cells. It controls glucose and nutrient delivery to the RPE and retinal cells, contributes to metabolic waste removal, and ensures a stable ionic environment which is critical to the optimal synaptic transmission among the retinal epithelial cells.

The RPE is a critical site of cellular injury that contributes to vision loss during AMD. Cells of the RPE degenerate due to environmental influences and multiple genetic perturbations (30). A well-established risk factor is gene polymorphisms that regulates both complement activation and lipid metabolism (31) but how they interact in the RPE to produce the observed pathologies remains poorly defined (32). Also, some individuals bearing polymorphisms associated with the disease never develop AMD indicating that other factors are involved in the manifestation of this visual impairment.

As currently understood, complement activation is a major driver of RPE loss, particularly so in AMD. Dysregulation of the complement system induces mitochondrial stress, fuels chronic inflammation and functionally damages the RPE (33). The pigment cells are both a target and a promoter of complement and these actions must be highly regulated to support ocular immune privilege. The most conspicuous mechanism related to complement -induced damage to the RPE is a result of the deposition of membrane attack complex (MAC or C5b-9) on the basal surface of the RPE. In retinal pigment cells this mediates sublytic stress, mitochondrial damage and enhanced inflammation which culminates in elevated ROS generation. The resulting ROS can be neutralized by locally produced melatonin when this system is intact but it is believed to be lost during aging thereby contributing to AMD.

MAC also triggers vascular epithelial growth factor (VEGF), a pro-angiogenic agent which advances local capillary growth (34). In wet AMD, the elevated production of vascular endothelial growth factor (VEGF) is the primary culprit in promoting choroidal neovascularization (35). VEGF secretion can be from the endothelial cells of the adjacent choroidal vessels, from macrophages, from activated T cells which are on-site because of the local inflammation, or from a variety of other cells in the vicinity. VEGF is often induced under conditions of low intracellular oxygen levels via the activation of HIF-1α; the activation of HIF-1α may be a result of elevated ROS which stabilize this transcription factor by activating prolyl hydroxylases (36, 37). In the retina of patients with myopic choroidal neovascularization, oxidative stress in the RPE promotes the growth of these vessels (38).

The pathophysiology of AMD also includes extracellular drusen deposits and intracellular lipofuscin accumulation in the retina. Lipofuscin, a term used to describe lipid peroxidation products in many different cell types, is composed primarily of vitamin A metabolites known as bisretinoid adducts in the RPE cells (39); lipofuscin accumulate in the lysosomes of the RPE cells when the outer photoreceptor outer segments are phagocytized (40). Because of the life-long recycling of the outer segments, the amount of lipofuscin bisretinoids continuously increase in the human retina, and this accumulation is accelerated when related genetic mutations exist (41). The bisretinoids interfere with normal RPE function, induce oxidative stress, and dysregulate homeostatic processes within the RPE cells to contribute to AMD.

Pathological yellowish extracellular accumulations of lipoproteins, referred to as drusen (Figure 1), form in the macula of the eye during both wet and dry AMD, with their number and size correlating with the advancement of this retinal disease (42–45). Drusen can form underneath (sub-RPE drusen) or above (sub-retinal drusen) the RPE cell layer. The more rapid advancement of sub-retinal drusen predisposes to the development of advanced AMD (46, 47). While much is known about the generation of lipofuscin-based bisretinoids, information related to the biogenesis of drusen is sketchy, especially regarding the genetic risk factors participating in their development and even whether they are the cause or a result of AMD (48). The formation of drusen involves free radical mutilation of critical molecules in both the dry and wet forms of AMD.

**Figure 2:**
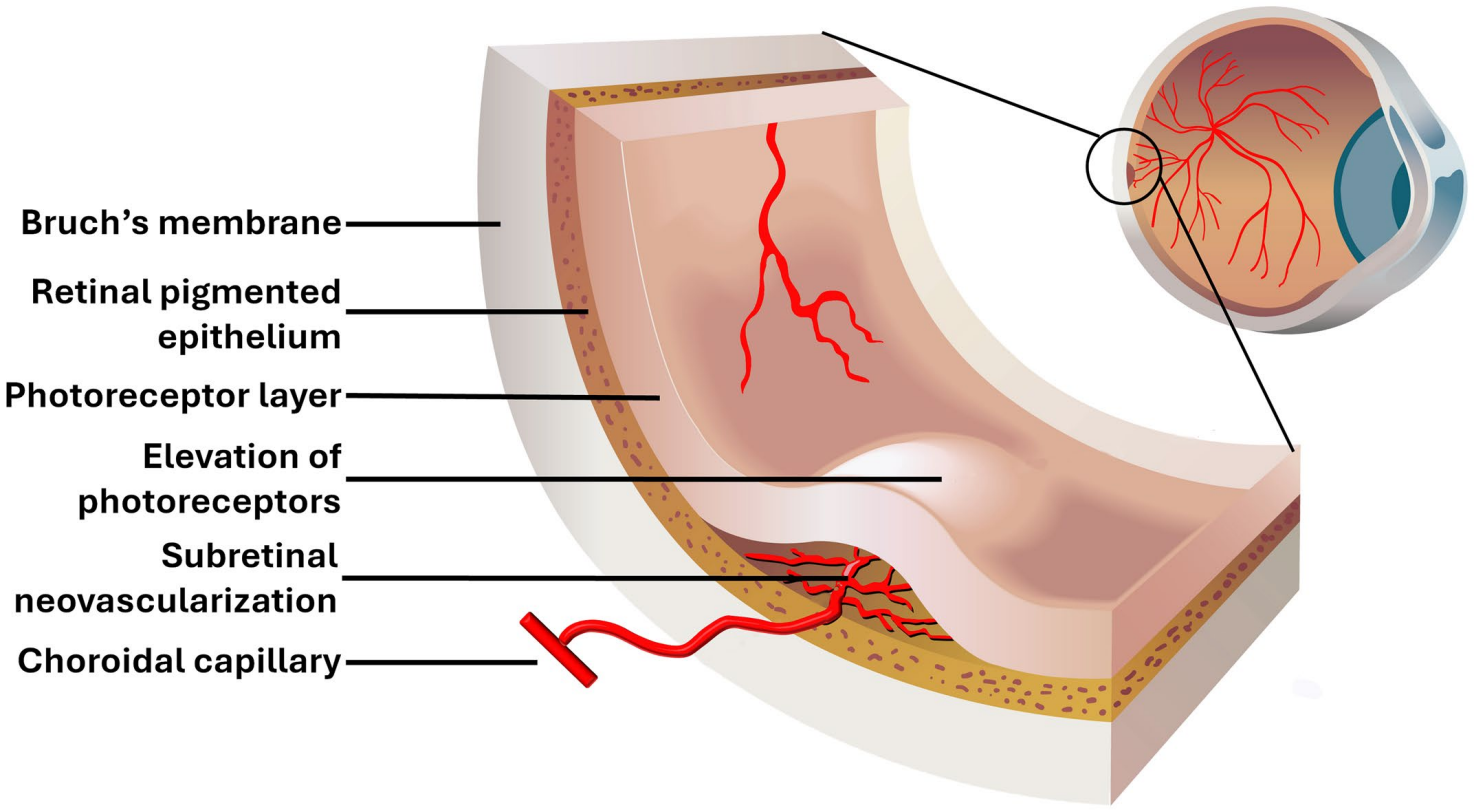
A major feature of wet AMD is the growth of blood vessels from the choriocapillaris through Bruch’s membrane in the macula. The new blood vessels can accumulate in either the subretinal space, illustrated here, or in the sub-RPE space. Also, these aberrant vessels become leaky causing edema. As the lesion expands, it causes the photoreceptor layer to buckle and degenerate which results in the loss of fine vision. Neovascularization is, in part, a consequence of excessive production and secretion of vascular endothelial growth factor (VEGF).

The pathological transformations of RPE cells in dry and wet AMD are distinctly different. While both forms involve destruction of tissue in the macula, the atrophic form of the disease (dry AMD) leads to progressive damage primarily due to the accumulation of oxidatively damaged molecules and thinning of the macula, ultimately impairing the function of the RPE. In its advanced stages, dry AMD is associated with the regional death of retinal cells, a sign known as geographic atrophy (49). The cell death, which often originates in the fovea, is primarily a result of oxidative damage and in addition to aging, it may be aggravated by high fat or high sugar diet and by smoking (50). Although less common, wet AMD can also exhibit geographic atrophy during its development. Additionally, wet AMD is associated with abnormal neovascularization under the macula by choroidal capillaries; these vessels often leak both fluid and blood cells, causing scar formation which leads to more rapid vision deterioration (51). The excessive growth of blood vessels is due to the increased production and release of VEGF (52). The new blood vessels breach Bruch’s membrane and form under the RPE or in the space between the RPE and the retina (subretinal space). This results in the loss of fine vision (Figure 2).

## 3 Oxidative stress and inflammatory responses in retinal pigment epithelium

While aging is considered one of the major contributing factors to AMD, the associated free radical-mediated damage to the RPE clearly plays a significant role in both forms of this ocular pathology (42, 53). This is because the retina, including the RPE, like most neural ectoderm-derived tissues, is highly oxygen-sensitive and critically dependent on a stable oxygen supply for its metabolism and function. Moreover, the photoreceptors are rich in polyunsaturated fatty acids (PUFA) which can be readily oxidized, with the damaged lipid products contributing toward mutilating additional PUFA. Also, proteins, DNA and carbohydrates are likewise harmed (54). The collective molecular disfigurement negatively impacts RPE mitochondrial physiology which further augments free radical generation (55, 56). Thus, over time, the persistent generation of damaging toxic oxygen derivatives impairs the function of the RPE which not only contributes to AMD but also causes damage to other ocular cells, leading to cataracts and development of glaucoma (57, 58).

**Figure 3:**
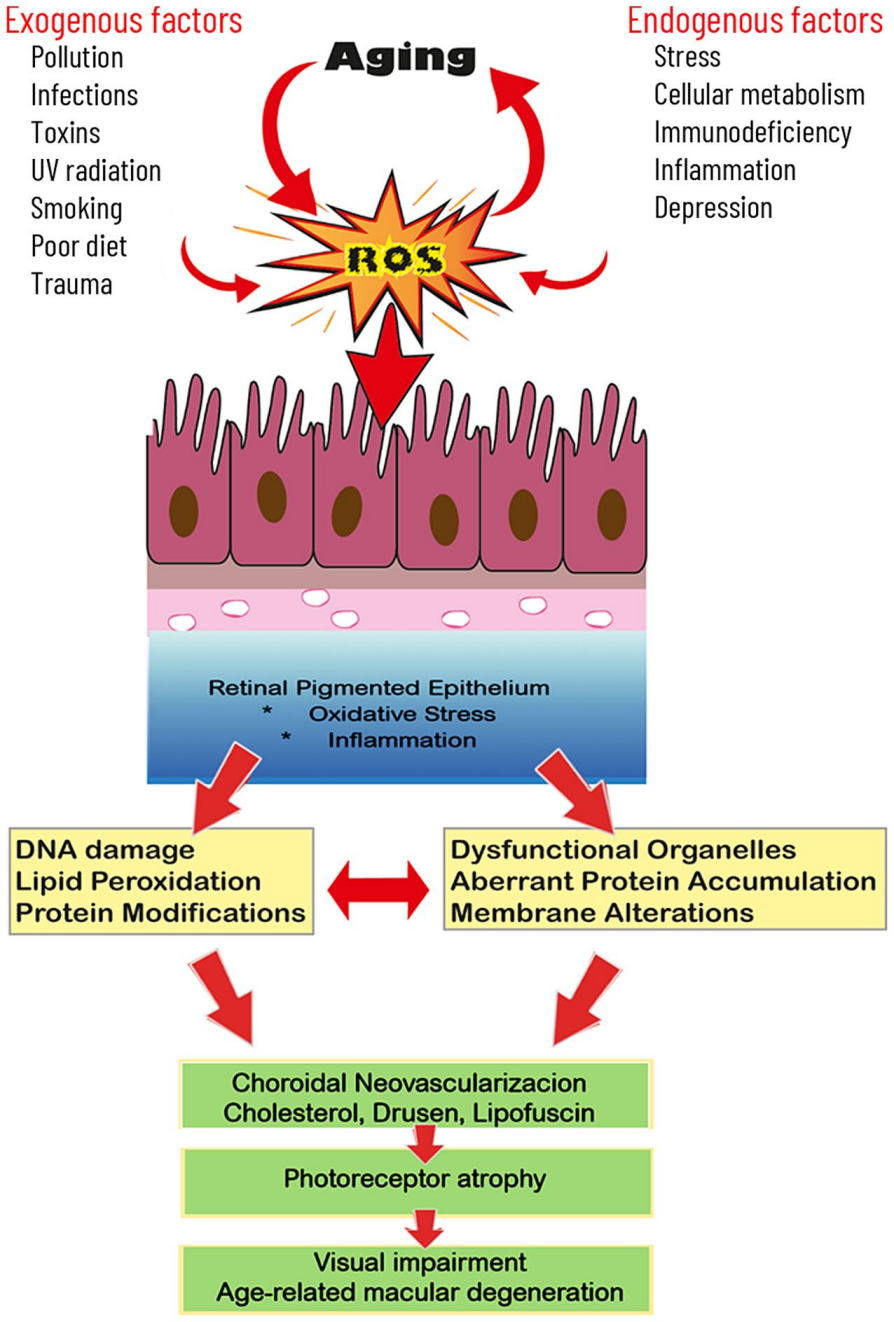
Aging and reactive oxygen species (ROS) reciprocally interact in the mediation of RPE damage which contributes to visual loss. A variety of extrinsic and intrinsic factors conspire to elevate ROS generation in the RPE cells. This leads to molecular damage to lipids, DNA, and proteins which culminate in cellular dysfunction. Since photoreceptor physiology is highly dependent on optimal RPE function, the disruption of the RPE cells contributes to photoreceptor atrophy and visual impairment.

Studies on post-mortem donor eyes of humans who suffered from AMD have verified the role of perturbed mitochondrial function and elevated oxidative stress to the RPE supporting the participation of these processes in the progression of these degenerative retinal diseases (Figure 3) (59). For example, the presence of 8-hydroxy-2-deoxyguanosine (8-OHdG), a known marker of DNA damage, is elevated in donor AMD eyes particularly when the RPE is atrophic (60). Other biomarkers of oxidative stress in human RPE of AMD patients include elevated levels of protein carbonyls and malondialdehyde, a common index of lipid breakdown (61). The elevated levels of oxidative stress parameters in the RPE are also associated with a depressed total oxidant status of the blood and a reduction of the antioxidant capacity in wet AMD patients (62, 63). For example, the antioxidative enzymes, superoxide dismutase (SOD) and glutathione peroxidase (GPx), as well as the indirect antioxidant, glutathione reductase, which converts oxidized glutathione back to its reduced form, were all depressed in the serum of individuals beyond 55 years of age (64). Likewise, SOD and both enzymes that metabolize hydrogen peroxide to oxygen and water, GPx and catalase, were lower in erythrocytes collected from AMD patients (65). The results of the human studies are consistent with those from experimental investigations which document a significant role for oxidative stress playing a major role in both dry and wet AMD (66).

Chronic inflammation is a component of many diseases associated with aging, such as Alzheimer’s disease (AD) (67). Although advanced age and oxidative stress are considered to be major triggers of retinal atrophy, AMD is a multifactorial disease in which inflammation also plays a conspicuous pathogenic role in both the dry and wet phenotypes (48). In dry AMD, chronic inflammation persistently upregulates complement pathways which amplify inflammation and associated tissue damage (68). The local inflammatory response supports the formation of drusen, contributes to degeneration of the RPE and adjacent photoreceptors, and disrupts Bruch’s membrane which, in wet AMD, allows the ingrowth of neovascular vessels into the subretinal and/or sub-RPE space (Figure 2). The inflammatory response is initiated by microglia/macrophages (type 1), neutrophils and dendritic cells and others which conspire to stimulate adaptative immunity including B cells and T cells. These cells secrete a host of inflammatory cytokines, e.g., interleukin-1β (IL-1β), IL-6, IL-8, transforming growth factor-β (TGF-β), interferon-*γ* (IFN-γ) and others (69, 70). Proinflammatory cytokines, especially IL-1β and IL-6 stimulate signaling pathways that activate NADPH oxidase, xanthine oxidase and mitochondrial free radical generation. The proinflammatory cytokines are usually used as defensive weapons to fight against invaders, e.g., bacteria, viruses, etc., that could otherwise inflict harm on the organism. If the processes are not interrupted, a vicious cycle ensues wherein the released free radicals promote the discharge of additional proinflammatory cytokines (71). Microglia and macrophages that infiltrate the area of the damaged retina are also participants in pathological retinal neovascularization in wet AMD (72).

## 4 Mitochondrial dysfunction in retinal pigment epithelium during age related macular degeneration

The highly specialized, polarized RPE cells are strategically placed between the choroidal capillaries plus Bruch’s membrane and the photoreceptors. They are essential caretakers of the photoreceptors because of their physiological interplay with photoreceptive elements and in the daily phagocytosis of their shed outer segments. Due to these latter activities, lysosomal and autophagic functions in the RPE cells are of major importance (73, 74). RPE cells also provide glucose to photoreceptors, recycle vitamin A and are essential components of the blood-retinal barrier. Like most neural elements, the RPE cells are mitotically dormant *in vivo* and, because of their many functional responsibilities, they are metabolically highly active, using mitochondrial oxidative phosphorylation (OXPHOS) as their major energy source (24). Because of their heavy burden of energy demands, mitochondrial homeostasis within the RPE cells is essential for ensuring adequate ATP production (75). Knockout of RPE-specific mitochondrial genes changes glucose processing in the cells, leading to photoreceptor degeneration (76).

**Figure 4:**
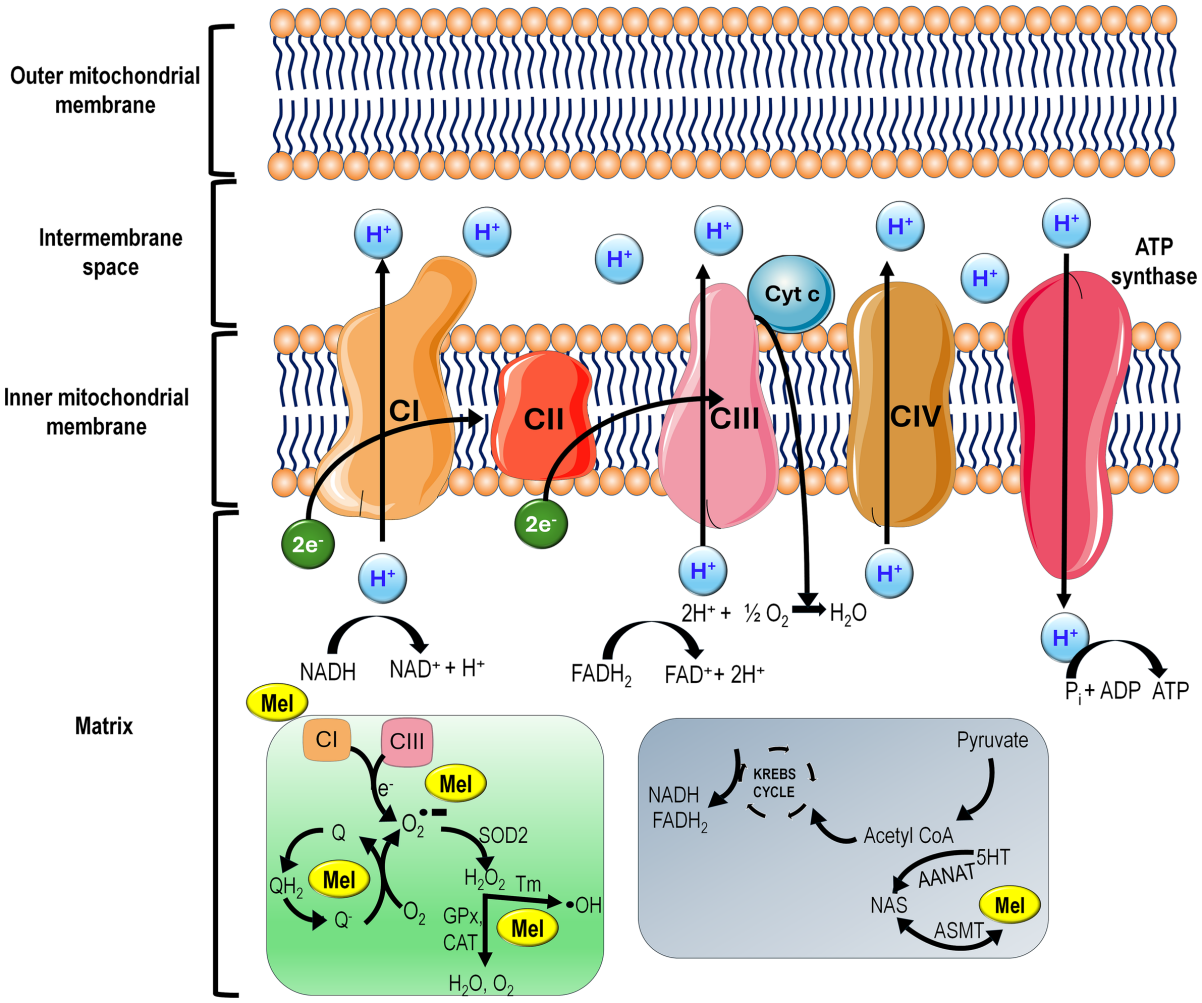
As discussed later in this report, melatonin is synthesized in the mitochondria and, therefore, presents in higher concentrations in this organelle than in other subcellular compartments. Shown are the transfer of electrons between the complexes of the electron transport chain. In the matrix, electrons (e^−^) escape from complex I (CI) and complex III (CIII) and help form superoxide anion radical (O_2_∙^−^), which is subsequently metabolized to other reactive oxygen species. Melatonin (MEL) located in the mitochondrial matrix scavenges each of these toxic reactants and stimulates antioxidative enzymes including mitochondrial superoxide dismutase (SOD2), glutathione peroxidase (GPx), and catalase (CAT) as indicated. The outlined scheme on the right illustrates the synthesis of melatonin in mitochondria. Acetylation of serotonin (5-HT) to *N*-acetylserotonin (NAS) by arylalkylamine *N*-acetyltransferase (AANAT) requires acetyl coenzyme (acetyl CoA), a product of pyruvate metabolism. Acetyl CoA also feeds into the Krebs cycle (citric acid cycle or tricarboxylic acid cycle) to generate the reducing agents NADH and FADH_2_ which provide e^−^ to the electron transport chain. NAS forms melatonin when it is methylated by acetylserotonin *O*-methyltransferase (ASMT). Both AANAT and ASMT are present in mitochondria and determine melatonin synthesis in these organelles.

Mitochondrial OXPHOS is continually in a state of change since the intramitochondrial oxidative environment fluctuates constantly between hypo- and hyper-oxygenated states. Even under normoxic conditions, OXPHOS generates free radicals (some of which are oxygen-based species) due to the leakage of electrons from the electron transport chain (ETC) when they are passed between the successive protein complexes (Figure 4). ROS production in the RPE cells becomes greatly exaggerated when intracellular oxygen levels deviate out of the normal range or if the proteins that constitute the ETC are damaged (77). The leaked electrons chemically reduce the ground state oxygen to the free radical, superoxide anion (O_2_^**∙-**^) (78, 79). The half-life of O_2_^**∙-**^ is brief and is either enzymatically dismutated by superoxide dismutase to H_2_O_2_ or it undergoes a diffusion-controlled reaction with nitric oxide (NO^**∙**^) to produce the peroxynitrite anion (ONOO^**−**^). The metabolic fate of ONOO^**−**^ is diverse; it can be enzymatically detoxified or it reacts with proteins, transition metals or carbon dioxide (80). Once formed, H_2_O_2_ can be removed by the action of glutathione peroxidase (GPx), catalase (CAT), or peroxiredoxin, all of which convert it to water and oxygen (81). However, when H_2_O_2_ encounters a transition metal such as divalent iron (Fe^2+^), it is non-enzymatically converted to the highly reactive hydroxyl radical (·OH). While this ROS species is extremely short-lived, it damages macromolecules in its close vicinity. Both ·OH and ONOO^**−**^ are very highly reactive and destructive to the RPE and photoreceptors, as has been shown to occur in AMD (66). In addition to being involved in the conversion of H_2_O_2_ to the ·OH via the Fenton reaction, iron dyshomeostasis in the retina aids in the development of AMD by activating the ferroptosis pathway, which leads to loss of the RPE cells and degeneration of photoreceptors (82).

Mitochondria, whose number varies among cells, are essential to the energy requirements of the cell. These critically important organelles participate in many cellular processes and are normally responsible for the majority of the ATP generated (83, 84). ATP synthesis via the ETC is a principal function of the inner mitochondrial membrane while the tricarboxylic acid (TCA) cycle and *β*-oxidation occur within the mitochondrial matrix. Mitochondria are highly dynamic organelles that can continually be renewed; when damaged, they are removed by mitophagy and fission (85). If mitophagy is interrupted, it leads to the accumulation of functionally impaired mitochondria which compromises cellular welfare (86). Both processes are essential to ensure mitochondrial quality control. Mitochondrial numbers in a cell are increased when a mitochondrion undergoes fission (87). The molecular requirements for fission to occur include the recruitment of dynamin related protein 1 (DRP1) to the outer mitochondrial membrane. Molecules that participate in recruiting DRP1 include mitochondrial fission factor and cardiolipin (88). Excessive levels of oxidative stress lead to mitochondrial damage and dysfunction such as in the RPE cells during AMD, causing cellular death. This is particularly serious in the RPE cells since they are mitotically inactive *in vivo* and are not replaced (89).

As previously mentioned, the retina, including the RPE, is highly oxygen- and glucose-dependent. Since oxygen is a common precursor of many oxygen- and nitrogen-based damaging free radicals produced in the mitochondria, these organelles and the cell within which they are located are vulnerable to oxidative stress (Figure 4). Due to their high metabolic demands, the RPE and other photoreceptors have an abundance of mitochondria (90). Oxidative stress accounts for many of the functional perturbations that the mitochondria within the RPE cells exhibit. Prolonged exposure to light radiation that enters the eye is one of the factors involved in ROS-mediated RPE damage (54). As in other cells, ROS can serve as signaling molecules for essential functions in the RPE cells (91). The exaggerated levels of ROS generation in the RPE cells, however, such as during ionizing radiation, ultraviolet radiation, poor life style including smoking, changes in intracellular oxygen concentrations, etc., overwhelms the mitochondrial antioxidant network leading to damage of vital proteins, lipids, and DNA (Figure 3) (92). The accumulated damage, if not repaired, eventually induces mitochondrial dysfunction and can trigger mitochondrial loss by mitophagy and cellular loss via autophagy. In damaged AMD cells, excessive production of ROS induces mitophagy through a process involving the p62/Nrf2 signaling pathway with this having a central role in AMD pathogenesis (93). A lower number of mitochondria has been observed in the RPE cells of AMD patients (94). Moreover, under stressful conditions, these actions are amplified in AMD since aging itself is associated with a reduction in endogenous mitochondrial antioxidant levels (95).

## 5 Age-related retinal degeneration and its relation to phase separation

**Figure 5:**
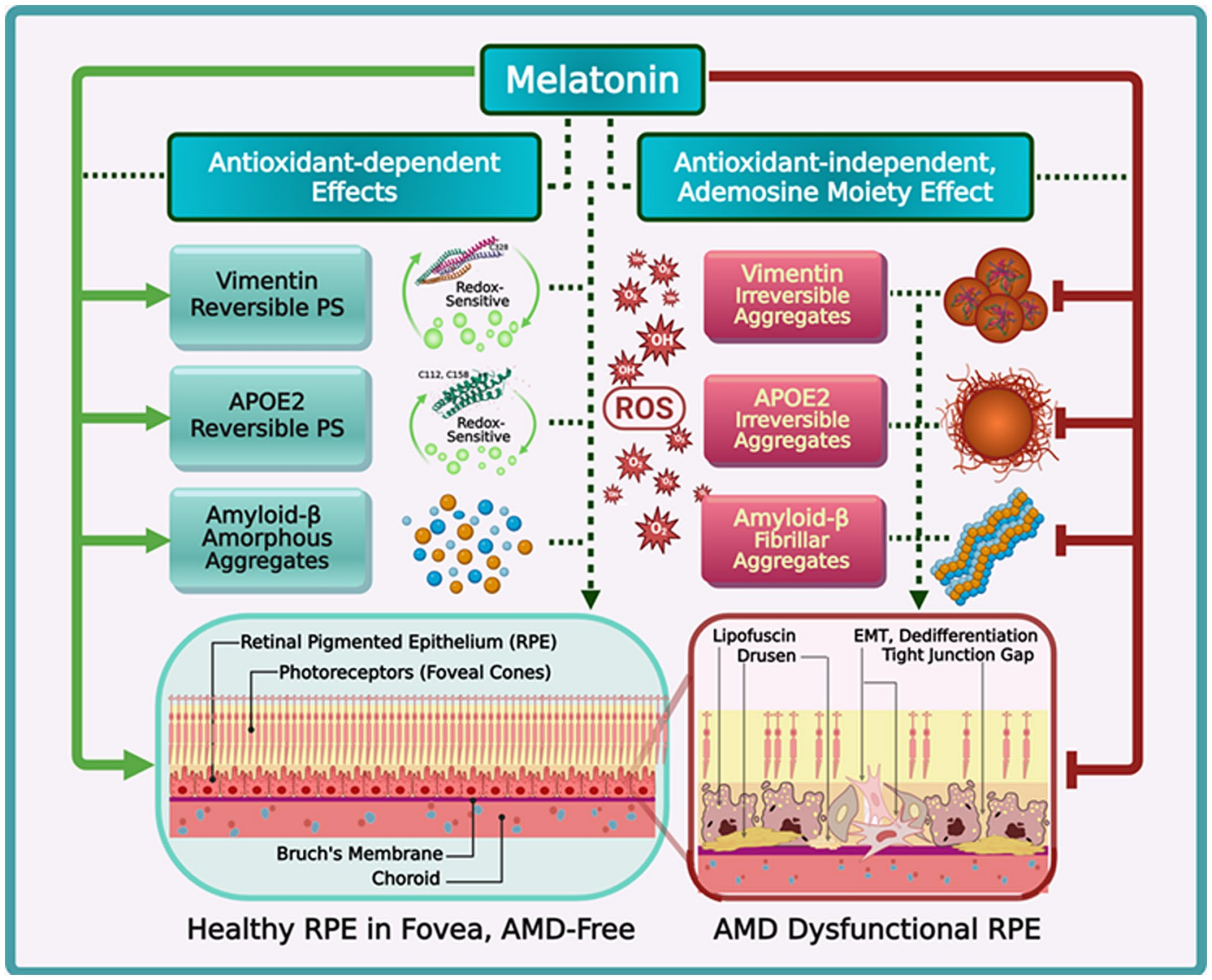
This figure depicts melatonin’s antioxidant-dependent and -independent mechanisms protecting against RPE dysfunction by regulating phase separation (PS). *Vimentin*: oxidation of C328 triggers reversible PS into biomolecular condensates (BCs), which reassemble upon stress removal, but prolonged oxidation leads to irreversible aggregates, promoting epithelial-to-mesenchymal transition (EMT) and RPE dysfunction, including drusen, lipofuscin, and tight junction gaps. *APOE2*: oxidation of C112/C158 enables reversible PS, but irreversible aggregation impairs A*β* clearance, contributing to drusen and lipofuscin. *Amyloid-β (Aβ)*: aberrant PS of Aβ promotes irreversible fibrillar aggregates, exacerbating RPE dedifferentiation and tight junction loss. Melatonin’s antioxidant effects neutralize oxidants, promoting reversible BCs formation via redox-sensitive oxidations of cysteines at position 328 (vimentin) and 112/158 (APOE2). Melatonin further augments the adenosine moiety effect via *π*-π stacking to solubilize irreversible aggregates of vimentin, APOE2, and Aβ, effectively attenuating EMT and AMD progression. Created with BioRender.com

The cells of all tested living organisms depend upon phase separation, a spontaneous thermodynamic process enabling fluid, reversible cellular compartmentalization, to rapidly organize/reorganize functions/reactions in response to the fluctuating cellular environment (96). A recent bioinformatic study that comparatively analyzed nine retinal proteomes and classified them on the basis of their specificity of gene expression and tissue distribution reported that proteins expressed in the retina exhibited increased propensity for phase separation due to elevated intrinsic protein disorder whereas broadly expressed proteins in multiple tissues were more ordered with higher structural stability (97). The dynamic interplay between the potential of proteins to phase separate and factors including oxidative stress as well as mutational burden may also contribute to the pathophysiology of RPE in AMD. The oxidative post-translational modification (PTM) of amino acid residues affects protein structure and functions (98). The reversible oxidation of a single cysteine residue in the *α*-helical rod domain of vimentin induces phase separation of reversible membrane-less condensates in its N-terminal low complexity domain (Figure 5). These droplets confer protection against filament depletion during oxidative stress. Consequently, the upregulation of vimentin is required for EMT (epithelial to mesenchymal transition)-mediated cancer metastasis (99). Similarly, in RPE cells exhibiting features of Type 2 EMT, vimentin was observed to be markedly upregulated (100).

Phase separation is now understood to drive the transition of amyloid beta (Aβ) from functional, intrinsically disordered, amorphous monomers into pathological oligomeric fibrils (101). The aggregation of Aβ oligomers is associated with neurodegenerative disorders including AMD (Figure 5) (102). Both Aβ and *α*-synuclein have been observed in drusen and the retina, respectively (103, 104). ROS can significantly elevate condensate viscosity, leading to the pathological aggregation of proteins associated with disease states (105).

The abundance of proteins in the retina with increased intrinsically disordered regions of proteins (IRDs) that promote the propensity for phase separation emphasizes both a heightened reliance upon phase separation and the need to manage the potential pathological effects of protein aggregation from phase separation. In addition to antioxidant-dependent features that can modulate redox-dependent phase separation processes, the proposed antioxidant-independent capacity of melatonin to regulate phase separation of condensates provides a unique perspective in appreciating the relevance of the local production of melatonin in the retina. Melatonin is reported in numerous studies to effectively inhibit EMT in tumorigenesis by targeting vimentin (106). It has also been observed to significantly reduce and suppress the aggregation of *α*-synuclein and Aβ in both *in vitro* and *in vivo* experimental conditions (107). By tuning the viscosity of condensates (108) and enhancing the adenosine moiety effect to protect protein hydration shells and prevent hydrophobic collapse often associated with amyloid fibrillization and aggregation (107), melatonin may be instrumental for the successful attenuation of AMD progression via the regulation of protein phase separation in the retina under normal and oxidative conditions.

During aging, mitochondrial dysfunction results in a vicious cycle wherein increased ROS production and decreased ATP levels result in aberrant phase separation of an extensive array of mitochondrial physiological processes (109, 110). The subsequent dysregulation of cardiolipin metabolism, chaperone functions, mtDNA transcription, fission, mitophagy, and proteostasis due to ATP deficiency-induced dysregulation of phase separation, further exacerbate mitochondrial dysfunction (111). Importantly, the biphasic regulation of phase-separated condensates by ATP is critical for maintaining the health of the RPE by promoting both optimal phase separation and preventing the aggregation of pathological proteins (112, 113). The three common human apolipoprotein E (APOE) isoforms, APOE2, APOE3, and APOE4, are implicated in the development of AMD as well as AD (114). The substitution of cysteine with arginine at positions 112 and 158 results in altered antioxidant capacity of the APOE isoforms in the order of E2 > E3 > E4 (115). Consequently, APOE2 (two cysteines) is associated with increased risk for AMD (Figure 5), but is protective for AD, while APOE4 (two arginine moieties, no cysteine) is protective in AMD, but is a strong risk factor for AD (114). It is plausible that the oxidation of cysteines in APOE2 affects lipid metabolism (116) leading to the dysregulation of cholesterol transport that contributes to the pathophysiology of AMD (117, 118). While previous attempts to validate a bidirectional relationship between AMD and AD yielded conflicting results that did not support an inverse relationship (119, 120), a bidirectional two-sample Mendelian randomization analysis identified a causal effect where early-onset AMD due to germline genetic variation reduced the risk for AD (121). Curiously, an early 2001 report found no association between AMD clinical phenotypes and the accumulation of APOE in drusen (122). Perhaps the redox-dependent phase separation of APOE2 explains the elusive molecular mechanism responsible for these controversial and puzzling observations. The upregulation of APOE as a result of mitochondrial dysfunction may be a compensatory metabolic response to mitigate RPE damage.

RPE cells are dependent upon mitochondrial OXPHOS to maximize energy production, suppressing glucose consumption to ensure adequate supply to the retina (123). Mitochondrial dysfunction in the RPE cells, therefore, necessitates increased aerobic glycolysis to produce adequate ATP for the suppression of potential aberrant aggregation of APOE2 condensates that can nucleate drusen (124, 125). In addition, the maintenance of proteostasis in RPE by molecular chaperones, including Hsp70, is also highly ATP-dependent (126, 127). The lack of adequate ATP production in older individuals may help explain why drusen are not found in subjects under 45 years of age, despite the presence of elevated APOE mRNA in the RPE. In advanced age (>75 years), AMD and drusen are present even though APOE mRNA levels in RPE cells are relatively lower as compared to younger ages (122). Hence, conditions that promote early AMD, such as the formation of APOE2 condensates in the absence of sufficient ATP from APOE4-induced glycolysis to prevent aggregation and nucleation of drusen, may become beneficial in AD. Unlike RPE cells, astrocytes are metabolically flexible (128) and neurons can easily switch to glycolysis (129) to ensure adequate ATP for maintaining proteostasis and preventing aberrant aggregation of elevated APOE2 condensates that also promote Aβ elimination. In RPE cells, the scarcity of ATP under mitochondrial dysfunction presents an additional challenge; thus, the ability of melatonin to support mitochondrial function in RPE cells by reducing oxidative stress (130) and augment the hydrating effects of adenosine via *π*-π stacking and van der Waals interactions (107, 124, 131) can significantly leverage the solubilizing effect of limited ATP availability to effectively suppress pathological protein aggregation and nucleation of drusen.

## 6 Melatonin as an antioxidant and anti-inflammatory agent

Melatonin has a host of cytoprotective actions which alter the course of AMD. Its antioxidant and anti-inflammatory effects are likely of special importance in deferring damage to the RPE. In neuroectoderm-derived cells, mitochondria themselves are capable of melatonin synthesis (14, 15). This has also been documented for cultured RPE cells (132). Moreover, considering that the mitochondria of every somatic cell in an organism is derived from oocyte mitochondria, and since it has been shown that the mitochondria of the female gamete produce melatonin, it is believed that this synthetic trait has been transferred to every mitochondrion in all cells (133, 134). Moreover, during early evolution the mitochondria of eukaryotic cells became the offspring of melatonin synthesizing prokaryotic proteobacteria (135–137). It is currently hypothesized that the ability of mitochondria to synthesize melatonin has been conserved throughout evolution such that the cells of every eukaryote produce melatonin in their mitochondria (20, 138, 139),

Melatonin synthesis in mitochondria puts it in a strategic location to neutralize the abundance of free radicals that are generated by this organelle, both due to leakage of electrons from the ETC as well as those produced because of enzymatic activities of oxidases, e.g., cytochrome c oxidase located in the inner mitochondrial membrane (Figure 4) (140). Evidence suggests that melatonin scavenges each of the reactive derivatives of oxygen that are produced in RPE cells, i.e., O_2_^**∙-**^, H_2_O_2_, ·OH, and ONOO^**−**^ (138, 141–147). Moreover, the metabolites that are generated when melatonin functions as a radical scavenger are equal to or better that melatonin in detoxifying ROS; some of these metabolites include cyclic-3-hydroxymelatonin (c3HOM), N-acetyl-N-formyl-5-methoxykynuramine (AFMK), N-acetyl-5-methoxykynuramine (AMK), 6-hydroxymelatonin (6OHM), 5-methoxytryptamine (5MT) and perhaps others (18, 19, 139, 147–154). As currently understood, the ability of these molecules to detoxify highly reactive species are chemical interactions that require no intervening receptors. Melatonin, however, also has receptor mediated actions that rid the intracellular environment of ROS. Membrane melatonin G-protein coupled receptors are common on many, perhaps all, cells (155–158) and when they bind melatonin, the activities of several antioxidative enzymes are stimulated, including SOD, GPx, glutathione reductase (GRd), CAT, and glutamate cysteine ligase (GCL), the rate limiting enzyme in glutathione production (21, 159, 160). Also, members of the retinoic acid-related receptor family (RORα, RORβ) may be involved in these processes (161).

Transition metals such as Fe^2+^ also are chelated by melatonin which reduces the formation of the devastatingly toxic ·OH species (162, 163), thought to account for more than 50% of the damage sustained by critical molecules during oxidative stress. Finally, melatonin repairs oxidatively damaged DNA which aids in preventing mutations (164–166) and it also inhibits telomerase which counteracts the shortening of telomeres, hexameric tandem repeats, on linear chromosomes during cell replication or during certain pathological processes (167). This was also shown to occur in RPE cells wherein melatonin prevented RPE telomere shortening resulting from streptozotocin toxicity (168).

Clearly, melatonin has at its disposal a plethora of direct and indirect means to quell oxidative stress and preserve lipids, proteins and DNA intact. These processes are caried out in RPE cells. Melatonin’s diverse and potent actions as a reducer of oxidative stress also have important bidirectional implications for regulation of autophagy in the RPE. The dysregulation of autophagic processes has been suggested to contribute to AMD (169). Primary cultured RPE cells markedly upregulated autophagy when exposed to chronic oxidative stress. Elevated oxidative stress was likewise associated with functional reduction of mitochondria, increased cell death and elevated accumulation of lipofuscin (170). These findings correlate with observations on donor human AMD cells wherein autophagic proteins, the number of autophagosomes in the cells, and cellular autophagy were reduced. Thus, chronic oxidative processes promoted autophagy while depressed autophagy reciprocally enhanced oxidative stress in RPE cells (171).

**Figure 6:**
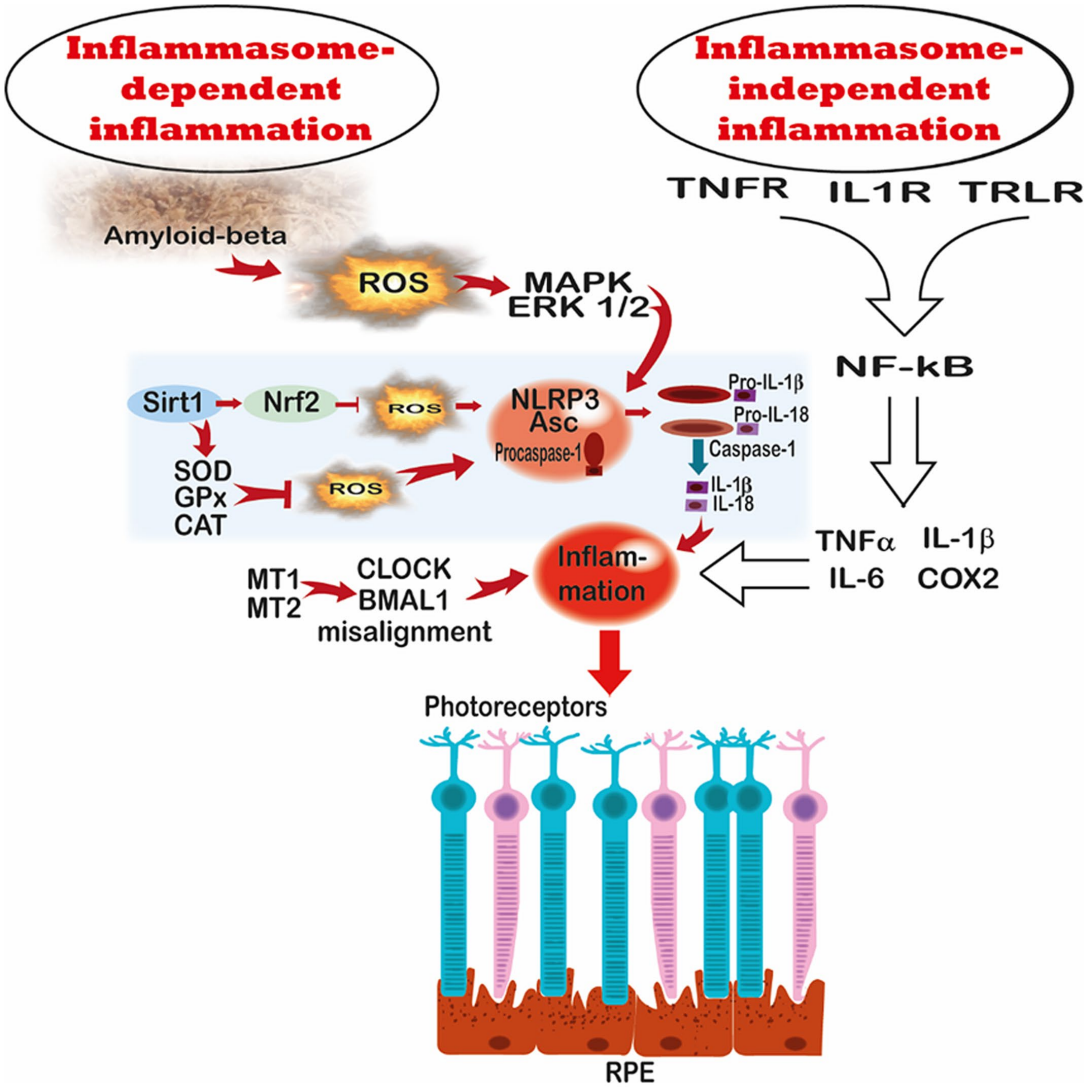
This figure summarizes some of the changes that occur during an inflammatory response in all tissues including those of the RPE. Both inflammasome-dependent and inflammasome-independent processes lead to inflammation and RPE dysfunction. ROS have multiple roles in stimulating the assembly of the NLRP3 inflammasome which induces inflammation. One of the master switches for inflammation is the transcription factor, NF-κB, which is normally sequestered in the cytosol in a bound state with IκB. It is activated through membrane receptors that respond to TNFα, IL-1, DAMPs, and other stimuli, leading to kinase-mediated phosphorylation of IκB and its subsequent degradation by the proteasome. The dissociation of NFκB/IκB allows for the translocation of NFκB into the nucleus where it binds to DNA and promotes the transcription of proinflammatory cytokines and other molecules that enhance the inflammation. Melatonin has multiple means by which it downregulates inflammation. By directly scavenging ROS generated in RPE cells, melatonin limits assembly of the NLRP3 inflammasome. Melatonin also upregulates SIRT1 which activates Nrf2, thereby enhancing the metabolism of ROS to less reactive or non-reactive molecules. Acting via melatonin membrane receptors (MT1 and MT2), it also upregulates antioxidative enzymes SOD, GPx, and CAT. By reducing the dissociation of NFκB from IκB, melatonin limits inflammasome-independent inflammation. Finally, by means of its interaction with membrane receptors, melatonin stabilizes circadian rhythms by regulating *CLOCK* an *BMAL1* gene expression. Regular circadian fluctuations are essential for optimal cellular physiology. Further description of these events is presented in the text.

Investigations of melatonin’s suppressive actions on inflammation have a long investigative history with these actions being summarized in numerous reviews (172–175). Mitochondrial inflammation, also known as mito-inflammation, occurs in several neurodegenerative diseases; this is an early response initiated by damage-associated molecular patterns (DAMPs). DAMPs activate receptors on the surface of cells that initiate several downstream cascades which result in the release of proinflammatory cytokines (176). Similarly, damaged mitochondria release mtDAMPs into the extracellular space which also triggers inflammation (177). mtDAMPs can include several different molecules such as ATP, cardiolipin, mtDNA and succinate; mtDNA is also an especially potent DAMP which directly activates the inflammasome (178) (Figure 6).

Melatonin’s anti-inflammatory actions have been investigated using many experimental models and endpoints (172, 175, 179, 180). Especially noteworthy is the ability of melatonin to inhibit the inflammasome, a critical driver of the inflammatory response in many diseases. By suppressing inflammasome assembly, melatonin limits inflammation via modulating pathways that relate to SIRT1, Wnt/*β*-catenin, long non-coding RNA and microRNA (Figure 6) (181). By upregulating SIRT1, melatonin also activates Nrf2 as well as downregulating NF-κB signaling while enhancing the release of IL-4 and IL-10, both of which function as anti-inflammatory agents; their actions attenuate the cytokine storm which is associated with an intense inflammatory response (182). Especially at the level of the mitochondria, melatonin’s antioxidant actions also come into play in deactivating ROS generated by dysfunctional ETC and by reducing pyroptosis (183). Additionally, melatonin controls macrophage polarization by preventing the formation of pro-inflammatory M1 macrophages (184, 185); retaining macrophages in a M2 phenotype is an important means by which melatonin quenches inflammation.

Amyloid β (Aβ) protein fragment is typically associated with neurodegenerative disorders, particularly in AD. The Aβ1-40 isoform is also a common component of drusen, indicating it is a contributory factor to AMD (186). Aβ upregulates NK-κB and inflammasome assembly, thereby inducing the generation of proinflammatory cytokines (Figure 6). At high concentration, Aβ induces RPE cell death suggesting a strong association between Aβ and RPE health (187); based on the accompanying molecular studies, the authors proposed that Aβ is a major factor that enhances choroidal neovascularization pathology. Since both AD and AMD develop in aged patients when endogenous melatonin levels are attenuated, it has been speculated that they are related diseases that occur in different tissues (188, 189). Moreover, given that melatonin is a well-known inhibitor of Aβ in the AD brain (190, 191), it is proposed that it would similarly inhibit Aβ formation in RPE cells and thereby alter AMD pathogenesis.

There are only a few publications specifically related to melatonin as an anti-inflammatory agent in RPE cells. The stimulus for one report was to define whether melatonin may mitigate diabetic retinopathy by suppressing inflammation in the RPE (192). Human RPE cells along with retinal endothelial cells were subjected to hyperglycemia by exposing them to D-glucose, a known promoter of proinflammatory mediators (193), or they were incubated with IL-1β. In this *in vitro* system, melatonin reduced the expression of inflammatory-associated cytokines including matrix metalloproteinase (MMP)-2 and MMP-9 which degrade the basement membrane allowing for the migration of neutrophils into the damaged tissue thereby exaggerating inflammation. Similarly, VEGF and intercellular adhesion molecule-1 (ICAM-1) were inhibited; these cytokines support inflammation by increasing endothelial cell permeability and enhance the adhesion and transport of immune cells into the damaged tissues which exaggerate the inflammatory response. The ability of melatonin to forestall the expression of each of these cytokines was considered as one means by which it reduces inflammation in the retina. These indirect findings support melatonin as an anti-inflammatory agent in RPE and cell in concordance with the ability of melatonin to protect against RPE cell degeneration (194, 195).

A second report utilized a mouse model in which retinal degeneration was induced following the administration of sodium iodate (SI) (86). In this system, necroptosis, which is commonly associated with inflammation, was elevated and NLRP3 inflammasome activation was upregulated (Figure 6). Melatonin mitigated these detrimental changes via the restoration of sarco/endoplasmic reticulum calcium ATPase 2 (SERCA2), an essential enzyme related to calcium signaling, and prevented mitochondrial calcium concentrations from reaching toxic levels leading to necroptosis. The protective actions of melatonin were negated when the animals were given a MT2 receptor antagonist, documenting that ability of melatonin to preserve RPE integrity after SI administration relied on the MT2/SERCA2/Ca2 + pathway (86).

A known contributor to inflammation in AMD is the stimulation of pattern recognition receptors (PRR) [including Toll-like receptors (TLR) and NOD-like receptors (NLR)] that recognize DAMPs and are common on innate immune cells (Figure 6) (196). DAMPs/mtDAMPs in the stressed retina are associated with drusen components such as amyloid *β*-oligomer as well as with RPE lysosomal damage (197), oxidative stress disfigured molecules such as mtDNA, and with the elevated concentration of advanced glycation end products (198–200). Although the ability of melatonin to depress the interaction of DAMP with PRR receptors on RPE or whether the associated inflammatory response occurs in other experimental models has not been examined relative to the RPE (201–203). It would, however, seem reasonable to have a similar protective effect in RPE cells where DAMPs are involved in the inflammatory response of this tissue (204).

Using an oxygen–glucose deprivation model as a representative of ischemia/reperfusion, Carloni and colleagues examined the effects of melatonin on mito-inflammation (205). In this system, melatonin proved highly protective against a number of pathological responses of mitochondria. By reducing oxidative damage, melatonin preserved the activities of complexes I, III, and IV of the ETC. It also limited the discharge of mtDNA into the cytosol resulting in a reduction in the release of proinflammatory cytokines, INFβ and IL6. The authors documented that melatonin, by protecting mitochondria from damage, proved to be a significant anti-inflammatory molecule, as also suggested in earlier reports.

## 7 Melatonin protects against retinal pigment epithelium dysfunction during age related macular degeneration

The antioxidant actions of melatonin are likely primary functions of this molecule in altering the onset and progression of both dry and wet AMD. Oxidative stress in multiple retinal cells certainly contributes in a major way to this devastating vision-impaired condition (26, 40, 206). As a multifaceted ROS/RNS direct scavenger, melatonin and its metabolic kin are significant factors in protecting against retinal destruction in AMD as well as in other retinal diseases, e.g., diabetic retinopathy and retinopathy of prematurity (207–209). Due to the antioxidant cascade in which melatonin and its metabolites successively neutralize ROS, the protective actions of this endogenously generated antioxidant are amplified; it is estimated that melatonin may scavenge as many as 10 radical species via this pathway (18). This is in marked contrast to classic antioxidants which generally scavenge a single radical. Additionally, the highest concentration of melatonin within a cell is in mitochondria where numerous ROS (an estimated 90% of the total ROS) are produced due to leakage of electrons from the ETC and their subsequent chemical reduction of ground state oxygen to O_2_^**∙-**^ (26). The latter is the precursor of a series of highly toxic oxygen derivatives, all of which are detoxified by melatonin (Figures 4, 6) (20, 21). Also, a central means by which inflammation causes molecular damage is a consequence of the production TNF-*α* and IL-1β which activate immune cells leading to the creation of damaging oxygen-based and nitrogen-based reactants (210). Furthermore, inflammatory cytokines trigger the activities of NADPH oxidases, lipoxygenase, xanthine oxidase and other oxidases which further enhance the number of ROS available for molecular mutilation (211, 212).

As in other tissues, ROS-mediated oxidative stress and inflammation-induced molecular damage in AMD is self-sustaining via a feedback loop that further exaggerates destruction of the RPE and consequently also of the photoreceptors. This occurs when excessive ROS interfere with normal ETC functions by damaging the carrier proteins (213, 214). This leads to the release of inflammatory mediators such as TNF-α and IL-6 which, in turn, recruit immune cells that additionally generate ROS, thereby enhancing the damage and propagating the feedback process (215). Transcriptomic analyses also indicate that elevated oxidative stress changes gene expression profiles in RPE cells, stimulating pathways that contribute to cellular senescence, angiogenesis and complement malfunction (216, 217). In view of the obvious role played by ROS in molecular damage that compromises RPE physiology and the reliance of rods and cones on healthy pigment epithelial cells, antioxidants have often been suggested as potential therapeutic agents for AMD (218, 219).

The pineal production of melatonin wanes with age in animals as well as humans (220, 221), which could be a major factor in the initiation and progression of AMD. The synthesis of melatonin in mitochondria is believed to follow the same dwindling pattern (140). Thus, RPE cells are left increasingly vulnerable to destruction which they sustain because of the elevated ROS that form in aged tissues (222, 223). The combination of the loss of the multifunctional antioxidant, melatonin, and the increased inefficiency of the ETC, which generates a steadily elevating number of free radicals in the aged RPE, may well contribute to AMD. This problem is compounded by the fact that RPE cells are mitotically inactive and once damaged, are incapable of being morphologically or functionally restored, inducing a downward spiral of their ability to support the overlying photoreceptors. As the damage accumulates, it bolsters drusen deposition and pathological neovascularization from the choroid (224, 225). Moreover, as AMD progresses, the release of pro-inflammatory cytokines enhances double strand breaks which lead to cell cycle arrest via a pathway that involves microRNA-23a and ROS. These phenomena emphasize the significant role that oxidative stress likely plays in mediating AMD (225) and suggests the potential use of melatonin to delay central blindness.

In addition to its efficacy in directly neutralizing ROS, a receptor independent process, melatonin mitigates the amount of oxidative damage accumulated pathologically within a cell by upregulating the Nrf2 (nuclear factor erythroid 2-related factor 2)/ARE (antioxidant response element) signaling pathway (226). This pathway involves Keap1 (Kelch-like ECH-associated protein 1) which is a sensor of oxidative stress (227) that binds to both the ubiquitin ligase (Cul3-Rbx1) and to Nrf2. Nrf2 is transcription factor that, after its separation from Keap, translocates to the nucleus where it forms dimers with Maf proteins and subsequently binds to the nuclear ARE for the transcriptional activation of critical cytoprotective enzymes that suppress oxidative damage and detoxify electrophiles (228). Melatonin upregulates Nrf2 by inhibiting its proteasomal degradation and by aiding its translocation into the nucleus (229). The action of melatonin in suppressing oxidative damage by modulating the Nrf2/ARE pathway has been documented in numerous experimental models and is widely accepted as a means by which melatonin controls redox homeostasis (230, 231). The regulation of the Nrf2/ARE pathway by melatonin is prevented by luzindole which blocks the membrane receptors for melatonin indicating that the actions on antioxidant enzymes are receptor mediated (232).

The role of Nrf2 in relation to AMD and the regulatory actions of melatonin have not been specifically addressed in RPE cells; however, given the numerous studies confirming the stimulatory actions of melatonin on the Nrf2/ARE axis accompanied by the upregulation of antioxidant enzymes in other cells, it is accepted that the same associations exist in the retinal pigment cells (233, 234). Without providing any molecular details, melatonin slowed morphological deterioration of the retina in the senescence-accelerated OXYS rat (235). In a more complete study in a rat model of dry AMD, melatonin treatment reduced RPE ultrastructural damage, and prevented changes in the choroidal capillaries as well as thickening of Bruch’s membrane. Also, the daily administration of melatonin maintained the melanin content of the RPE and reduced melanosome loss with the results being consistent with the use of melatonin as a promising antioxidant agent to combat dry AMD (236).

Melatonin has been sparing examined in reference to inflammation in the RPE (237). However, considering melatonin’s thoroughly investigated anti-inflammatory actions in other tissues, it is expected that melatonin would suppress inflammatory responses in RPE cells as in other tissues. The processes involved include the inhibition of the activation of NLPR3 inflammasome by suppressing the release of pro-inflammatory cytokines, e.g., IL-1*β*, and determining the polarization to macrophages to the M2 phenotype, which is anti-inflammatory (Figure 6) (238, 239). Moreover, its ability to detoxify ROS obviously contributes to its anti-inflammatory actions. Melatonin also modulates pathways such as Wnt/β-catenin, SIRT1, long non-coding RNA and micro/RNA and others which are functionally related to immune regulation and inflammation (181, 240).

Telomerase dysfunction in RPE cells may also be a feature of AMD which contributes to cellular senescence (241). Impaired telomerase function supports the inflammatory state of the RPE by enhancing NF-κB activity which contributes a redox imbalance resulting in increased cellular damage and senescence. Melatonin regulates telomerase activity by influencing its catalytic subunit of hTERT (human telomerase reverse transcriptase) (167) in many cell types; it is speculated to upregulate this enzyme in RPE cells as well (242, 243) to preserve cellular activity and reduce cell death, important for the post-mitotic RPE cells.

## 8 Melatonin synthesis in the eye

Although melatonin is best known for its circadian synthesis and release from the vertebrate pineal gland, this is by no means the only organ that produces this important indoleamine (244). Current evidence suggests that there are two sources of melatonin in vertebrates with distinctly different functions (140). Because of its exclusive nocturnal production in the pineal gland, its subsequent release from this gland is responsible for the circadian rhythms of melatonin in the blood and cerebrospinal fluid (CSF), with the latter seemingly having a greatly exaggerated rhythm relative to that in the blood. It is presumed that the daily fluctuation in circulating CSF melatonin concentrations assists in the regulation of bodily circadian rhythms via an interaction with clock genes in the suprachiasmatic nucleus (SCN) of the hypothalamus. Moreover, the day-night changes in blood melatonin levels presumably cue clock genes in peripheral cells (158, 245). Thus, the pineal production and secretion of melatonin is key for the maintenance of circadian biology which is unequivocally indispensable for cellular and organismal health (246). The actions of melatonin in the regulation of both the master and peripheral biological clocks requires its interaction with membrane melatonin receptors (247).

With the exception of retinal photoreceptors, the synthesis of melatonin in extra-pineal tissues neither exhibits circadian changes nor is it exported into the blood. Locally produced melatonin contributes to the maintenance of cellular health due to its antioxidant, anti-inflammatory, and other intracrine, autocrine and paracrine actions (205, 248). For at least some of these functions, an interaction of melatonin with receptors is not required. In all tissues, the intracellular production of melatonin primarily occurs in mitochondria (14, 15). This location is important since many ROS are generated in this organelle and, furthermore, mitochondria are involved in numerous other critical functions, all of which are impacted by melatonin (111, 249–251).

With reference to the eye, there is one *in vitro* study that documented melatonin production by RPE cells (132). Additionally, several other ocular tissues synthesize melatonin including photoreceptor, lens, iris, and ciliary body (252–254). Also, classic melatonin membrane receptors have been identified in the photoreceptors, cornea, choroid and sclera (255). In the rods and cones but not in other ocular tissues, melatonin is generated in a circadian manner as in the pineal gland (253). As is characteristic for all extrapineal cells, melatonin synthesized in ocular tissues is not released into the blood but is rather used locally (140). Melatonin influences several circadian rhythms in the eye including the regular shedding of photoreceptor disks, amelioration of elevated intraocular pressure, influencing cataract physiology, and impacting neuronal sensitivity (256, 257). While RPE cells produce their own melatonin intrinsically, it is not known if photoreceptor-derived melatonin influences the function of the RPE cells, for example, by protecting them from oxidative damage. This seems highly probable considering the morphological and functional intimacy of these two ocular tissues. This would be consistent with the paracrine actions of retinal circadian-released melatonin which also impacts dopamine synthesis by amacrine and interplexiform cells as well as by synchronizing outer segment disk shedding (258, 259).

If ocular melatonin synthesis diminishes with increased age, it could be a contributing factor toward AMD. While there is no definitive evidence documenting a drop in melatonin production in the aging RPE cells, in the pineal gland where melatonin has been most thoroughly investigated relative to age, it exhibits a marked reduction in old rodents (220) and with the evidence showing that this also occurs in the human pineal gland (221, 260–262). These changes coupled with the increased ROS generation in aged RPE cells along with the reduction in the activities of antioxidative enzymes would constitute a situation conducive to the onset of AMD (222, 223).

## 9 Melatonin and AMD in humans

The initial clinical study related to melatonin and AMD was conducted by Yi and colleagues; both dry and exudative subjects were investigated (263). In this case-controlled study, 50 patients given melatonin (3 mg daily) for 6 months showed reported retardation in the progression of fundal pathology, with only 14 subjects (8 with retinal bleeding and 6 with more retinal exudate) exhibiting a worsening condition. Considering the outcome of this study, it could be assumed that the ability of melatonin to reduce the progression of AMD is modest. While the low dose of melatonin (3 mg daily) and a relatively short duration of treatment (6 months) exhibited positive effects, it may not have been sufficient to markedly influence AMD pathology (263). Based on thorough allometric calculations, it has been estimated that the most effective doses for inhibition of disease processes in the human would be between 1.0–1.5 mg/kg body weight (264).

A recent multicentered retrospective study involved thousands of inpatient and outpatient subjects in the US who either had no evidence of AMD at the onset of the investigation or were previously diagnosed with the condition (23). These individuals were queried as to their melatonin use and placed into either the melatonin group or the control group. The authors reported that patients who used melatonin exhibited a reduced likelihood of developing AMD as well as a slower progression of the disease in those who had been diagnosed as having AMD. There was little information on the doses of melatonin or whether its intake was daily or intermittent. Also, the duration of melatonin use was not well defined. Nevertheless, the consistency of the evaluated outcomes relative to both reduced disease development and retardation of the advancement were emphasized by the authors as supporting the conclusion that melatonin may be useful as a treatment for AMD. They also surmised that the efficacy of melatonin for inhibiting AMD likely involved its ability to reduce oxidative stress of the RPE along with known inhibition of VEGF (23).

Despite strong support from experimental studies, the evidence of melatonin’s ability to alter the onset or progression of human AMD is inadequate to make a definitive judgment about the significance of daily melatonin use in resisting this devastating condition. Based on the amount of melatonin marketed globally per year (estimated to be $2.8 billion in 2025), there are thousands of individuals who take melatonin on a regular basis, likely over a very large dose range, who could be retrospectively studied (23). The endpoints in these investigations could consider the progression of a number of chronic diseases that accompany aging. When these studies are carried out, it is essential that the patients be stratified on the basis of the daily dose of melatonin with information on the duration of treatment being carefully cataloged. In consideration of the calculations of (264), the low doses of melatonin (3–5 mg daily) that are often used for sleep modulation would likely be insufficient to significantly inhibit any serious age-related chronic disease including AMD. This is also an opinion shared by the authors of the current report.

## 10 Conclusion

AMD is a complex disease of ocular tissue, especially of the macula, which is driven by multiple molecular, environmental and genetic factors. The two subtypes of AMD, i.e., dry and wet, have some different pathophysiological features. Dry AMD is characterized by the deposition of lipid and protein pools, referred to as drusen, underneath the RPE adjacent to Bruch’s membrane. This pathological feature interferes with the essential movement of nutrients and waste products between cells of the RPE and photoreceptors. This can culminate in geographical atrophy which are accumulations of atrophic RPE and photoreceptor cells causing loss of vision (265). The most characteristic feature of wet AMD is the growth of blood vessels from the choriocapillaris into the retina. This is a result of the aberrant secretion of excess VEGF by epithelial and other cells in the area. The new capillaries that invade the retina are fragile and release fluid into the intercellular space resulting in localized macular edema which eventually causes scare formation and loss of fine vision (266).

Oxidative stress, inflammation, VEGF and phase separation are primary contributors to the pathologies of one or both dry and wet AMD. The RPE and photoreceptors are highly metabolically active tissues as reflected by the above normal numbers of mitochondria present in them. Excessive light exposure induces ROS generation in the retinal tissue that can exceed the antioxidative capacity of these cells culminating in the accumulation of oxidatively damaged macromolecules which compromise vision. Other risk factors that promote oxidative damage of the retina include smoking, obesity and hypertension, all of which are responsible for oxidative stress and inflammation (267).

**Figure 7:**
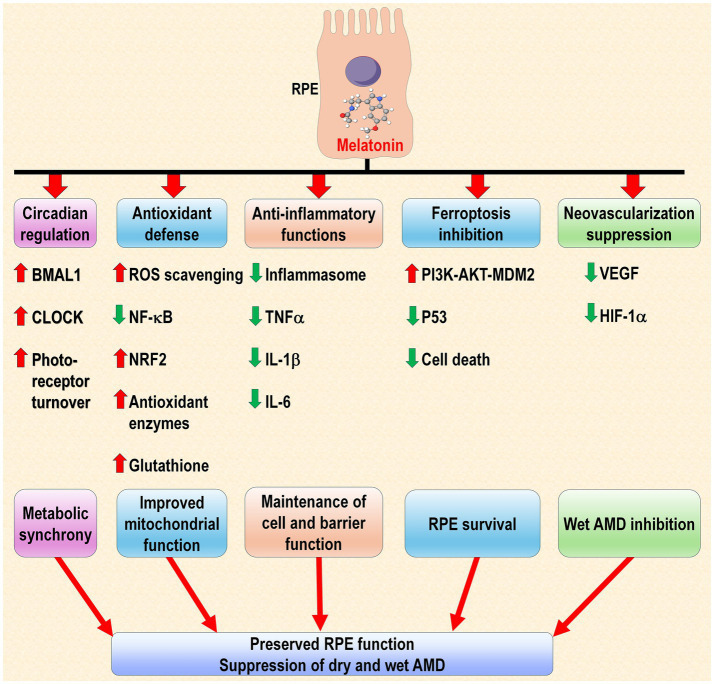
This is a summary of some of the processes in the RPE that melatonin modulates which could potentially delay the onset of AMD pathology or the progression of the disease. Melatonin, which is synthesized in the RPE, as well as that released from adjacent ocular tissues may be a factor in preserving the functional integrity of the pigment epithelial cells. These cells play a crucial role in supporting the functions of the overlying photoreceptors so maintaining RPE welfare is a key process for the maintenance of optimal vision. With the exception of neovascularization, which relates exclusively to the wet form of the disease, the other processes identified may be involved with both the wet and the dry forms of AMD. The circadian regulation of CLOCK and BMAL1 in RPE could be a function of the pineal-derived blood melatonin rhythm or from the photoreceptor-derived, which is also released in a circadian manner. Melatonin levels diminish with age concurrent with the development of AMD. Green arrows indicate inhibition or downregulation, and red arrows indicate stimulation of upregulation.

The major actions of melatonin that protect retinal tissues from damage are likely its antioxidant, anti-inflammatory, regulation of phase separation, and anti-VEGF functions. Its ability to limit ROS-mediated damage and suppress immune responses are applicable to both dry and wet AMD. Many reports have suggested the use of antioxidants as a potential means to reduce AMD (268). The anti-VEGF actions of melatonin which have been repeated confirmed in pathological conditions (269) have applicability to wet AMD; anti-VEGF drugs are commonly prescribed to combat this form of the disease (270). The multiple beneficial actions of melatonin which could support its use as an anti-AMD drug are summarized in Figure 7.

As currently recommended, melatonin is typically taken once per day about 30–60 min before desired sleep time. This means that for large portions of each 24-h period, vulnerable cells may be less defended since the half-life of melatonin in the circulation is about 40 min (271, 272). A more stable elevated level of melatonin may prove more effective as a treatment of inflammatory or oxidative stress-related conditions such as AMD (273–275). The translation of this information to the human is supported by the evidence showing that melatonin is likely an important and useful molecule to modify the course of AMD and since it is inexpensive and has a very large safety profile in terms of dose (no LD50 has been identified despite attempts to do so), it should be seriously examined as a protection against AMD.

Medical research directed toward age-related diseases is especially important in today’s society. For several reasons, the average life span of humans has increased significantly over the last half century. In 1975, the average life span in the US was 72.6 years; that number has increased to 79.6 years in 2025. This progressive rise in life span is predicted to continue in the foreseeable future. This being the case, the number of individuals afflicted with age-related diseases including AMD will become an increasing societal burden. If means are not found to forestall these debilitating conditions, the number of families that will have to adjust their lifestyle to accommodate the affected family member will likewise rise and grossly overburden welfare systems.

## Glossary

5-HT: 5-hydroxytryptamine
5-MT: 5-Methoxytryptamine
6-OHM: 6-Hydroxymelatonin
8-OHdG: 8-hydroxydeoxyguanosine
AANAT: Aralkylamine N- acetyltransferase
Aβ: Amyloid β peptide
Acetyl CoA: Acetyl coenzyme A
AD: Alzheimer’s disease
AFMK: N1-Acetyl-N2-formyl-5-methoxykynuramine
AMD: Age-related macular degeneration
AMK: N1-Acetyl-5-methoxykynuramine
Apo-E: Apolipoprotein-E
ARE: Antioxidant response element
ASMT: Acetyl serotonin O-methyltransferase
DAMP: Damage-associated molecular pattern
DRP1: Dynamin-related protein 1
EMT: Epithelial to mesenchymal transition
GPx: Glutathione peroxidase
GRd: Glutathione reductase
H_2_O_2_: Hydrogen peroxide
HIF-1α: Hypoxia inducible factor-1 alpha
iBRB: Inner blood retinal barrier
ICAM-1: Intercellular adhesion molecule 1
IFN-γ: Interferon γ
IκB: Inhibitor κB
IL: Interleukin
IRDS: Intrinsically disordered regions of proteins
Keap1: Kelch-like ECH-associated protein 1
MMP: Matrix metalloproteinase
NF-kB: Nuclear factor kB
NLRP3: NOD-like receptor pyrin domain containing protein
NO: Nitric oxide
Nrf2: Nuclear factor erythroid 2-related factor 2
NRL: NOD-like receptor
O_2_^-^: Superoxide anion radical
oBRB: Outer blood retinal barrier
OH: Hydroxyl radical
ONOO^−^: Peroxynitrite anion
OXPHOS: Oxidative phosphorylation
PRR: Pattern recognition receptor
PS: Phase separation
PTM: Post-translational modification
PUFA: Polyunsaturated fatty acid
ROS: Reactive oxygen species
RPE: Retinal pigment epithelium
SERCA 2: Sarco/endoplasmic reticulum calcium 2 ATPase
SIRT1: Sirtuin 1
SOD: Superoxide dismutase
TCA: Tricarboxylic acid cycle
TGF-β: Transforming growth factorβ
TLR: Toll-like receptor
VEGF: Vascular endothelial growth factor
